# Examining worldwide postmarketing suicides from biologics used for psoriasis with a focus on brodalumab: A cross-sectional analysis using the Food and Drug Administration Adverse Event Reporting System (FAERS)

**DOI:** 10.1016/j.jdin.2022.08.010

**Published:** 2022-08-27

**Authors:** Samuel Yeroushalmi, Mimi Chung, Erin Bartholomew, Marwa Hakimi, John Koo

**Affiliations:** Department of Dermatology, University of California, San Francisco, San Francisco, California

*To the Editor:* Biologics are highly efficacious biologic for treating moderate-to-severe plaque psoriasis, particularly brodalumab.^1^ However, the use of brodalumab has been severely limited due to a black box warning and an associated Risk Evaluation and Mitigation Strategies program indicating a possible increased risk of suicidality. This warning was applied due to 4 possible suicides during phase III clinical trials, of which only 3 were verified. Closer examination of these cases shows that all 3 had significant life stressors and no chronological relationship between drug administration and suicide was present.^2^ Given that brodalumab has been approved for nearly half of a decade, we aimed to examine worldwide postmarketing data to evaluate the association between brodalumab use and risk of suicide compared to 10 other biologics approved for psoriasis.

The total number of completed suicides for each biologic was extracted from the Food and Drug Administration Adverse Events Reporting System (FAERS) for all indications included through December 31, 2021.^3^ The FAERS is a publicly available international database for reporting postmarketing drug adverse events. The total number of patients prescribed each drug for all indications was determined by contacting pharmaceutical companies.

The number of total completed suicides for each biologic agent and the number of completed suicides per total patients prescribed are shown in Table I and Fig 1. Two suicides were reported for brodalumab on the FAERS. One suicide was reported in the United Kingdom but was deemed fraudulent due to a lack of mandatory reporting details required by the British Dermatology Association and British nursing agencies. The only verifiable suicide on brodalumab occurred in a Japanese male in his 50s with metastatic stage 4B lung adenocarcinoma and lived alone with no nearby relatives. He committed suicide 36 days after his first dose of brodalumab, and it remains unknown whether he self-administered follow-up doses.

**Table I:** Total completed suicides, total patients prescribed, and suicides per total patients prescribed for all indications for each biologic

Biologic name	Completed suicides since approval	Total patients prescribed	Corresponding date for total patients prescribed	Suicides per total patients prescribed
Tildrakizumab	0	n/a	n/a	n/a
Risankizumab	0	n/a	n/a	n/a
Brodalumab	1∗	20,871	July 31, 2021	4.79 × 10^−5^
Ixekizumab	4	175,000	February 28, 2021	2.29 × 10^−5^
Guselkumab	4	n/a	n/a	n/a
Certolizumab	12	n/a	n/a	n/a
Ustekinumab	12	n/a	n/a	n/a
Secukinumab	17	>500,000†	December 23, 2021	3.20 × 10^−5^†
Etanercept	62	n/a	n/a	n/a
Infliximab	84	3,100,000	August 01, 2020	2.61 × 10^−5^
Adalimumab	175	>1,400,000†	January 01, 2020	1.04 × 10^−4^†

The total number of completed suicides are reported for each biologic as of December 31, 2021 as reported by the FAERS. The total number of patients prescribed worldwide for each agent was obtained by directly reaching out to pharmaceutical companies. The corresponding date for the most recent value for the total number of patients prescribed is also listed. When calculating suicides per total patients prescribed, the number of completed suicides up to the corresponding date that was provided by pharmaceutical companies was used in order to account for differences in reporting from each pharmaceutical company. Values which were unable to be obtained are indicated with “n/a”.

∗ Investigation from the parent pharmaceutical company of brodalumab found that there were 2 reports of suicide: one in Japan and one in the United Kingdom (UK). The reported case in the UK, however, was deemed fraudulent due to a lack of details from corroborating organizations such as mandatory reporting from nursing agencies and reports from the British Dermatology Association, despite multiple outreach attempts. Therefore, we only included one verifiable suicide from Japan for brodalumab for this report.

† The value of n could only be provided as a lower estimate for adalimumab and secukinumab and so for the purposes of this report, this lower estimate value was used to calculate suicides per total patients prescribed.

**Fig 1:**
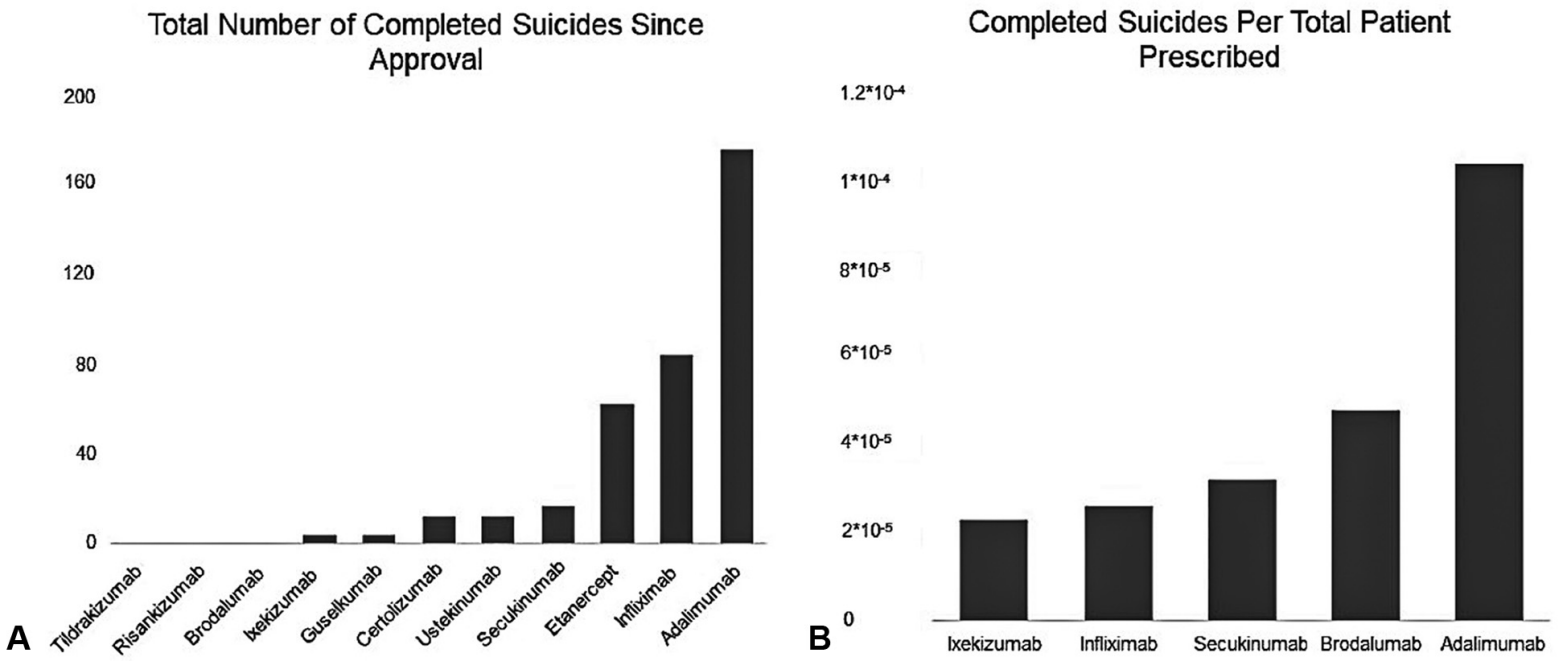
**A,** Total number of completed suicides for all indications for each biologic since approval as of December 31, 2021 and (**B**) the number of suicides per biologic per total patients prescribed for drugs for which data were available. Values are reported in Table I.

When comparing the total number of suicides reported and suicides per total patients prescribed since approval, brodalumab appears similar to other common biologics like adalimumab. Worldwide postmarketing data revealed only one confirmed suicide in a patient with metastatic cancer and little support system. Additionally, the unknown dosing of brodalumab in this patient fails to provide strong evidence of a causative relationship. Considering that postmarketing data are often considered a better reflection of real-world outcomes than clinical trials (conducted in more structured settings), the available worldwide data do not support the notion that brodalumab has a unique risk of increased suicides.

In addition to reporting bias from using FAERS data, limitations of this study include the fact that the total number of patients prescribed biologics is mostly the best estimates provided by pharmaceutical companies and that some of these values were not available at all despite the authors’ best efforts to obtain them. Moreover, FAERS data do not provide granular details such as preexisting risks, duration of drug use, and other details on the patient data. Despite these limitations, these novel results using the FAERS database highlight the possibility that brodalumab may not increase risk of suicidality and that further investigation is warranted.

## Conflicts of interest

J Koo is an advisor/consultant/speaker for Ortho Dermatologics, AbbVie, Boehringer Ingelheim, Celgene, Eli Lilly, Janssen, LEO, Merck/Sun, Novartis, Regeneron, Sanofi, and EPI Pharmaceutical Corporations. The remaining authors have no conflicts of interest to disclose.
